# Prevalence and Risk Factors for the Presence of Gastric Ulcers in Pleasure and Breeding Horses in Italy

**DOI:** 10.3390/ani14121806

**Published:** 2024-06-17

**Authors:** Sara Busechian, Francesca Bindi, Simona Orvieto, Francesco Zappulla, Maria Chiara Marchesi, Irma Nisi, Fabrizio Rueca

**Affiliations:** 1 Department of Veterinary Medicine, University of Perugia, Via San Costanzo 4, 06126 Perugia, Italy; maria.marchesi@unipg.it (M.C.M.); irma.nisi@acmedrugs.com (I.N.); fabrizio.rueca@unipg.it (F.R.); 2 Department of Veterinary Sciences, University of Pisa, Viale delle Piagge 2, 56124 Pisa, Italy; francesca.bindi@phd.unipi.it; 3 Independent Researcher, 06100 Perugia, Italy; simona.orvieto@gmail.com; 4 Azienda Unità Sanitaria Locale (AUSL) Umbria 2, Viale Bramante 37, 05100 Terni, Italy; fr.zappulla@hotmail.it; 5 Sport Horse Research Center, Department of Veterinary Medicine, University of Perugia, Via San Costanzo 4, 06126 Perugia, Italy

**Keywords:** Equine Squamous Gastric Disease, Equine Glandular Gastric Disease, pleasure horses, breeding horses, risk factors

## Abstract

**Simple Summary:**

Equine Squamous Gastric Disease and Equine Glandular Gastric Disease identify lesions in the two mucosae of the stomach. Different prevalences have been found in horses of different breeds engaged in different activities. Risk factors for the disease have been identified in some populations, with few studies evaluating pleasure horses. A cohort of 316 animals was sent for a gastroscopy to evaluate the presence and severity of lesions in either the mucosa or the stomach. A questionnaire about signalment, management, and health of the horses was given to the owners to identify possible risk factors for the two diseases. The prevalence of lesions of the squamous mucosa was similar to other reports and associated with signalment and management of the horses, especially the time and type of paddock, hay, and supplementary feed administered and time spent with the current owner or caretaker. Being used as a breeding animal was also a significant risk factor for ulcers in the squamous mucosa. In this population, the percentage of horses presenting lesions of the glandular lining was lower than in other studies, and of the parameters evaluated, they were associated only with the signalment of the horse, especially age and sex; management had a minimal effect on the development of ulcers in this part of the stomach.

**Abstract:**

Equine Squamous Gastric Disease (ESGD) and Equine Glandular Gastric Disease (EGGD) are two terms used to indicate the presence of lesions of the squamous and glandular mucosa of the stomach. Prevalences, pathophysiology, and risk factors are different, and the latter have been investigated in different populations. The aim of this study was to investigate the prevalence and risk factors of ESGD and EGGD in a cohort of pleasure, breeding, and retired horses in Italy. To the authors’ knowledge, this is the first study to investigate such a diverse population of animals and the first one that includes a large number of animals in Italy. Gastroscopies were performed in 316 animals, with and without clinical signs of gastric ulcers, and a questionnaire about signalment, management, activity, and health was given to the owners or caretakers. Prevalence of ESGD was similar to the current literature reports in comparable populations, and the disease was associated with signalment, time with the current owner or caretaker, management (time and type of paddock, hay, and supplementary feed administered), and activity performed. In this population, EGGD was present in a lower percentage of animals and, of the parameters evaluated, was associated only with the signalment, while management does not seem to influence the development of lesions in the glandular mucosa in this population.

## 1. Introduction

Equine Gastric Ulcer Syndrome (EGUS) is a worldwide disease that affects the stomach of equids. A recent consensus statement divided the disease into two different entities, one affecting the squamous mucosa (Equine Squamous Gastric Disease, ESGD) and one affecting the glandular lining (Equine Glandular Gastric Disease, EGGD) [1,2,3,4,5,6]. The two illnesses have different prevalences, pathophysiologies, and treatments. ESGD and EGGD have been investigated extensively in Thoroughbreds and Standardbreds in active training, with some small studies focusing on other groups of sport horses, such as those used for elite endurance competitions, polo, and Western performance, but also engaged in university riding programs and historical horseraces. Other studies have tried to investigate the prevalence of the disease in pleasure horses in Poland, in Thoroughbred breeding mares at pasture and in donkeys in Italy, and in feral horses. The percentage of animals affected in these populations is variable but generally lower than in racehorses, ranging from about 40% to around 70% [3,4,5,6,7,8,9,10]. While risk factors for ESGD and EGGD have been identified for racehorses [1,2,3,11], less information is available about horses not subjected to heavy training loads, especially breeding animals. According to the literature, in these populations, an effect of the development of gastric lesions has been found for breed, age, temperament, stabling, diet, and amount of exercise [1,2,3,12,13,14]. ESGD is considered to be caused by exposure to gastric acid in portions of the stomach that are normally not in contact with it, and possible risk factors for it are related to the management and intensity of exercise [3,5,6]. EGGD, on the other hand, is associated with a decrease in the protective mechanisms of the glandular mucosa, with stress and pharmacological treatments as the main causes [3,4,6]. The aim of this study was to determine the prevalence and risk factors for ESGD and EGGD in a group of horses used for low-intensity exercise, breeding, or retired from sports activity. To the authors’ knowledge, this is one of the first studies to investigate a diverse cohort of animals from a breed, age, and exercise point of view, and it is the first to evaluate horses in Italy.

## 2. Materials and Methods

### 2.1. Horses

Horses at least two years of age were included in this retrospective study. At the request of the referring veterinarians or the owners, animals were subjected to a gastroscopic examination to determine the presence and severity of gastric ulcers. If possible, all subjects present at a single facility were examined, irrespective of the presence of clinical signs compatible with gastric ulcers. An oral informed consent was obtained from the owners. Exclusion criteria: (1) treatment with gastroprotective drugs, either currently or in the three months before the examination; (2) involvement in heavy training and long-distance traveling (elite showjumpers, racehorses, etc.). Breeding mares were not pregnant at the time of the endoscopy, and weaning of their foals had been conducted at least 3 months before the examination.

### 2.2. Gastroscopy

Gastroscopy was conducted according to the literature [1,15] using a portable processor (Tele Vet X Led, Karl Storz Endoscopy, Tuttlingen, Germany) and a 3 m scope (60130PKS, Karl Storz Endoscopy, Tuttlingen, Germany). The horses were sedated with xylazine (0.5–1 mg/kg IV) and restrained with a twitch. After inserting the scope through the ventral meatus of the right nostril, it was advanced through the upper respiratory tract and the esophagus until it reached the stomach. The organ was inflated with air, and, if necessary, the surface was cleaned with purified water passed through the working channel of the endoscope so that it was possible to evaluate both mucosae. The presence and severity of the lesions of the squamous mucosa were recorded and graded according to the indications of the ECEIM Consensus Statement, and the horse was considered positive for ESGD for grades of at least 2/4 [1,16]. Alterations of the glandular mucosa were recorded and described based on the indications of the ECEIM Consensus Statement (presence, anatomical localization, distribution, and appearance). Due to a lack of a recognized scoring system for EGGD and of any indications about the clinical significance of the lesions of the glandular mucosa, for statistical purposes, animals were considered positive for any change in the surface of the mucosa [1,2,4,16]. Videos of the endoscopies were recorded and stored for evaluation and analysis.

### 2.3. Questionnaire for the Caretakers

A questionnaire about management, activity, and health status was given to the owners before the endoscopy (see Supplementary Material Table S1). Questions were based on current recognized risk factors based on the literature [3,4,5,6,17] and on the interview about history and signalment used at the University Veterinary Teaching Hospital of the University of Perugia. Overall, 2 open and 23 multiple choice questions were divided into different sections: signalment (breed, age, sex, temperament, and time spent with current caretaker), management (bedding, paddock, number of feedings per day, type of hay, and supplementary feed fed), activity (type of exercise performed, if any), time spent training per week and traveling per month, if any, history of heavy training in the past), and health (last dental check, concurrent treatments, abnormal behaviors, history of colic episodes, stable vices). A final open-ended question was included, where the caretakers could add comments about their horses. The answers were coded and recorded, together with the results of the gastroscopy, on an Excel sheet for analysis.

### 2.4. Statistical Analysis

Descriptive statistics were performed to determine the prevalence of ESGD and EGGD in the population. For statistical analysis, age groups were formed: young (below 10 years of age), adult (11–20 years old), and old (21 years old and above). Breeds were also grouped: Blood horses (e.g., Thoroughbreds, Arabians, Standardbreds, Quarter Horses, etc.), Saddlebreds (Italian, German, Dutch, etc.), Baroque horses (Friesians, Lipizzaners, etc.), Coldblood horses (Italian Draft horses, Haflingers, etc.), and Ponies. Normality was verified using the Shapiro–Wilk test. The correlation was determined using Spearman correlation coefficients. Simple and multiple logistic regression were performed to determine the effect of the different items of the questionnaire on the presence of ESGD and EGGD. Logistic models are used to evaluate the effect of predictor variables on the presence of a disease. The probability of the outcome is transformed into a logarithm, and it is modeled as a linear function of the predictors. From the models, odds and odds ratios can be calculated [18]. In this study, multiple models were built, including all questions present in each of the four sections of the questionnaire (signalment, management, activity, and health status), excluding the ones where collinearity was present (Spearman correlation results above 0.4). For the section evaluating the health status, two additional models were created, one considering the intensity and treatment of colic signs and one on the behaviors detected by owners, to focus on the presence of the symptoms most commonly related to gastric ulcers in the literature. Odds ratios (OR) and confidence intervals (CI) were calculated for each model. The goodness of fit of each multiple model was determined using ANOVA. Statistical significance was set at *p* < 0.05. Statistical analysis was performed on RStudio 2023.12.1 [19].

## 3. Results

Overall, 316 horses were included in this retrospective study. The population was aged between 2 and 28 years (median 10 years, interquartile range 6–14 years), with 169/316 (53%) classified as young, 124/316 (40%) as an adult, and 23/316 (7%) as old. Blood horses were 149/316 (47%), Saddlebred 144/316 (46%), Baroque horses 14/316 (4%), Coldblood horses 2/316 (1%) and Ponies 7/316 (2%). A total of 43/316 (14%) animals were stallions, 185/316 (58%) were females, and 88/316 (28%) were geldings. According to the owners, 238/316 (75%) horses were calm, and 78/316 (25%) were nervous (Table 1). Animals were recruited mostly from Central Italy, with some coming from the South and the North. Management systems were similar across the whole country, so the horses evaluated could be considered representative of the population in Italy.

All owners answered all questions, even if sometimes an explanation had to be given by the interviewers. Most of the horses had been living with their current caretakers for at least 2 years, were stabled in a box (91/316, 29% did not have a stable available), and had access to a paddock or pasture for at least some time during the day (124/316, 39% did not have access to a paddock). Most of the animals were fed 2 to 3 times per day (94/316, 30% and 113/316, 36%, respectively), with grass hay as the main source of roughage (269/316, 85%) and some kind of commercial supplementary feed (20/316, 6% were not fed any sweet feed). Around half of the horses were used for breeding (99/316, 31%) or did not perform any kind of exercise (64/316, 20%), and the rest were mostly used for English riding activities, especially show jumping (64/316, 20%). When exercised, they were ridden mostly every day at low intensities (walk and trot, 53/316, 17%). Of the 316 horses, 273 (86%) did not travel or were shown at any competition. Almost three-quarters of the animals (226/316, 72%) had never performed any kind of heavy exercise in the past. Sixty percent of the horses (186/316) did not have a dental check while with their current caretaker, and only 3/316 (1%) were treated with medications, either inhaled corticosteroids for asthma or pergolide for pituitary pars intermedia dysfunction (PPID). Ninety-one percent of the horses did not show colic signs while in their current stable (289/316), and the ones that did presented low-intensity symptoms that either did not require any treatment or responded to only symptomatic therapy with either flunix meglumine or N-butylscopolammonium bromide, with or without fluid therapy. Recurrent colic signs were present in 6/316 (2%) of the animals. Between 96% and 99% of the equids did not present changes in behavior (while feeding or otherwise) or stable vices. Responses and the distribution of the answers according to the presence of ESGD and EGGD can be found in Table 1.

Results of the Spearman correlation analysis showed a weak or low relationship between all questions except between age and age group and between time in paddock and type of paddock used, where there was a very strong correlation between the questions (rho 0.83 and 0.88, respectively, *p* < 0.001).

The presence of ESGD was recorded in 114/316 (36%) of the horses. In particular, grade 2/4 affected 60/316 (19%) of animals, grade 3/4 was found in 28/316 (9%), and grade 4/4 in 26/316 (8%) horses. Two hundred and two/316 (64%) animals were negative: grade 0/4 was identified in 134/316 (42%) and grade 1 in 68/316 (22%). According to the simple models, risk factors for ESGD were age, both as a number and considering the age group, breed groups, temperament, time the horse spent with the current caretaker, type of paddock, and time spent in it each day. Also, lesions of the squamous mucosa were determined by the times the animals were fed, either roughage or supplementary feed, each day, the type of hay and supplementary feed administered, the activity performed by the horse, and the time spent exercising each week. Finally, the time since the last dental check of the horses seems to have an effect on the development of gastric ulcers of the squamous mucosa in this population. The multiple models, on the other hand, showed that ESGD was influenced by signalment, especially breed groups, temperament, and time with current owners, but also by management (type of bedding, paddock and time spent in it, type of supplementary feed used) and activity (type and time spent exercising). The health section of the questionnaire did not influence the development of ESGD, except for the time since the last dental check. OR, CI, and p-values of the statistically significant simple models were reported in Table 2, while the results of the multiple models are presented in Table 3.

EGGD was identified in 32/316 (10%) horses as areas of hyperemia of the mucosa along the greater curvature. According to the simple models, EGGD was influenced by the age, both as a number and as a group, of the horses, by their breed and sex, and also by the time with the current caretaker. Furthermore, type and time in the paddock, number of feedings per day, and type of hay were found to be risk factors for lesions of the glandular mucosa. Lastly, the type of activity and time spent exercising each week, as well as the time since the last dental checkup, determine the presence of EGGD. Considering the multiple models, signalment (age groups, sex, and time with the current caretaker) and time since the last dental checkup determined the presence of lesions of the glandular mucosa. The OR, CI, and p-values of the statistically significant simple models can be found in Table 4. The results of the multiple models are presented in Table 5.

## 4. Discussion

ESGD and EGGD are quite common diseases in the equine population worldwide, with different prevalences found in the literature based on the type of exercise and management [1,2,3,4,5,6]. The highest percentage of horses positive for ESGD or EGGD has been found in racehorses [1,2,3,4,5,6], but, in a study, around 70% of Thoroughbred mares at pasture were affected [9]. High prevalence was also described in high-level showjumping horses [20] and long-distance endurance animals [21]. A recent study in pleasure horses in Poland found EGUS in almost 40% of animals showing no signs of gastrointestinal problems, but the prevalence was higher (60%) when clinical symptoms compatible with gastric ulcers were present [10]. Risk factors have been identified for different categories, from racehorses [3,4,5,6] to animals presented to referral hospitals [13,20], to Icelandic horses [12], to wild equids [22], but scarce information is available in the literature about pleasure, breeding, and retired animals. The aim of this study was to determine the prevalence of ESGD and EGGD in horses considered not at risk for gastric ulcers and identify risk factors for the presence of the two diseases in Italy.

In this study, ESGD was present in 36% of animals, comparable to other populations of horses not subjected to heavy training loads, such as asymptomatic pleasure horses in Poland [10], warmblood showjumpers in Canada [20], and horses in Italy [17]. The prevalence found for ESGD in this study was lower than those found in Thoroughbred mares at pasture [9], in non-athletes and intact males in Italy [23,24], in pleasure horses in Denmark [14], and in show animals in the United States [25].

The prevalence of EGGD in this population (10%) was lower than previously reported in different populations, such as Warmblood showjumpers [13,14,20], polo horses [26], and racehorses [11]. Before the publication of the ECEIM Consensus Statement in 2015 [1], though, the prevalence of gastric ulcers was reported for both mucosae at the same time, with few studies giving indications of the lesions of the two mucosae separately: comparing prevalences reported in older studies is, therefore, not always possible. The severity of the disease is also difficult to compare because of the lack of a validated grading system [1,2]. In this study, all horses presented only hyperemia of the mucosa without evident ulcerations or erosions. The clinical significance of EGGD is still not completely elucidated, despite the fact that some studies have found a correlation between the disease and racing below expectations in Thoroughbreds [2,3,4,11]. No information is currently available about pleasure horses and Saddlebreds.

Considering the simple logistic models, risk factors for the presence of ESGD and EGGD in this population are age, breed, time with the current owner/caretaker, access to a paddock and time spent in it, number of feedings per day, type of hay and supplementary feed administered, type of activity performed, time spent exercising each week, and time since the last dental checkup. Temperament was associated only with ESGD, and sex was associated only with EGGD.

The effect of signalment on gastric ulcers is controversial in the literature. Increased time in training is associated with higher grades of lesions, and racehorses older than 3 years were found to be more affected by the disease in various studies [1,3,4,5,6,27,28]. In a university riding program, younger and older animals seem more at risk of ESGD compared to the other age groups [5,29]. In the population investigated in the current study, animals aged less than 10 years are at increased risk for ESGD and EGGD. This could be explained by changes in management that could happen in the earlier years, with animals being moved to start training or being sold. Older horses, on the other hand, have a more stable environment, especially when more than 20 years of age, and are usually retired from exercise and active competition.

The breed is not considered a risk factor for ESGD in the literature [3,5], while sport horses and Warmblood are at increased risk for EGGD [4]. In the cohort evaluated in the present study, blood horses, such as Arabians or Thoroughbreds, are more at risk than Saddlebreds. The first are usually more nervous than the latter, and this could explain their predisposition to developing gastric ulcers. A recent study showed that the response of cortisol to ACTH stimulation was higher in Thoroughbred and Warmbloods compared to French–Montagne, indicating an effect of breed on the stress levels of horses [30].

Male horses are at increased risk for EGGD in this population: stallions are usually kept separated from other horses, do not have access to a paddock, and could be more nervous than other horses, all considered risk factors in this study but also in others in the literature [2,3,4,6,11].

Temperament was found, in the present study but also in others in the literature [3,5,25], as a possible risk factor for ulcers of the squamous mucosa: stress has been implicated in the development of gastric lesions [3,5], and nervous animals are probably more stressed than calmer subjects, also in this population of pleasure and retired horses studied in this paper.

No access to any kind of paddock has been related to ESGD and EGGD in various papers [4,6,12,20,26], and in the population in this study, turnout in a grass paddock and for at least some time during the day seems to be protective for the development of lesions of the squamous, but also of the glandular mucosa. The type of paddock was not evaluated in other publications, but high prevalences of gastric ulcers have been found in Thoroughbred mares at pasture [9]. In another publication, the pH of the stomach was not influenced by stable or paddock housing [31], so the exact effect of the turnout on gastric health is not completely understood. The effect of housing on stress levels in horses has been found to be quite controversial in the literature, with various studies suggesting a beneficial effect of individual stables and others suggesting a detrimental effect of the same conditions [30]. A recent study demonstrated that moving the horses to single housing from pasture can be an intense stressor, inducing stress-related immunomodulation with changes in the number and relative percentage of white blood cells [32].

These studies could also explain why, in the horses studied in our paper, the time with the current owner/caretaker is related to the presence of gastric ulcers: changes in management, stabling, social interactions, and traveling could all be stressful situations for the animals, leading to the development of lesions. These parameters were considered when creating a questionnaire to determine the risk of horses developing EGUS [17]. Despite not being correlated with ESGD or EGGD in the population evaluated in that study, changes in social interaction and travel were factors the owners were asked to keep in mind when determining the risk class for their animals.

The number of feedings per day, especially of sweet feed, has also been one of the questions in the survey previously reported [17]. In the population studied in the present paper, though, the higher the number of feedings, the higher the risk of developing EGGD. This could be explained by the fact that the amount of supplementary feed was higher in horses fed three or more times compared to the others. These animals were also generally subjected to higher levels of exercise and exercised for longer times each week compared to others in this cohort, parameters found as risk factors both in the present study and in the literature [1,3,5,6,11,17].

In the population investigated in the present paper, as can be found in other studies [3,5,6,11], training for more than 6 h per week has been found to be related to ESGD and EGGD. Exercise has been found to be related to gastric ulcers since the beginning of the studies on the disease. The increase in intraabdominal pressure caused by the movement pushes the acidic content of the stomach towards the cardias, exposing more of the less protected squamous mucosa to the low pH and increasing the risk of ESGD [33]. Another study found that gastrin levels increased after training. This hormone is responsible for the release of gastric fluid, and this could be another mechanism that relates to exercise and gastric ulcers [34].

In this study population, the type of hay administered is related to the presence of lesions in both mucosae. Supplementing grass during the winter seems to be protective for ESGD: these horses are kept in a grass pasture, which in this study has been associated with a decreased risk of gastric ulcers. This type of stabling is more similar to the physiological management of horses, which, in nature, eat for most of the day to counteract the continuous production of gastric acid [5]. In the present study, in horses with lesions of the glandular mucosa, feeding alfalfa was associated with an increased risk of disease. This is contrary to a recent study, where pelleted alfalfa was found to be protective for EGGD [35]. Another study, though, found that feeding alfalfa as chaff in weanlings increased the number of lesions in the pylorus area compared to feeding pellets; both formulations did not improve the score of the gastric lesions [36]. In the population investigated by our group, alfalfa was administered as forage, not pelleted, and no information was available about the amount of time the plant had been used before the gastroscopy. It is possible that the owners decided to change the diet when the horses started showing signs that they thought were compatible with gastric ulcers. The appearance of the roughage was not investigated, nor was the amount of alfalfa fed recorded, so it is not possible to exclude that the chaffs could have increased the risk of EGGD, even in this population of adult horses and where the lesions were located in the greater curvature. Further studies are needed to evaluate the effect of alfalfa on gastric health.

In our population, the type of supplementary feed is a risk factor for ESGD. Mixed feeds (pressed and pelleted together) and pressed ones increase the presence of ulcers in the squamous mucosa. This could be caused by the increased availability of sugar and starch that various studies have associated with lesions of the mucosa [3,5,6,37]. It has been demonstrated that changing diet to ad libitum hay and a low-starch supplementary feed can improve or heal ESGD even without treatment with omeprazole, but the amount of complementary feed administered (1 kg) in that study was lower than what is normally used in competition horses, even at low levels of exercise [38].

Breeding is a risk factor for ESGD in these horses. In the literature, this factor is not investigated quite clearly. Breeding Thoroughbred mares at pasture have a high prevalence of gastric ulcers, but no information is available about the cause of this finding [9]. Results about the possible stress levels in mares and stallions are variable in the literature. In mares, transrectal ultrasound increased salivary cortisol and heart rate variability parameters [39], while in stallions, semen collection has only a slight transient effect on stress levels [40]. In the population in this study, both mares and stallions were evaluated, and it was seen that sex has no effect on the development of ESGD; breeding has a negative impact on the squamous mucosa of both female and male horses.

Activities labeled as “other” (i.e., draft, Western, and show) increased the risk of gastric ulcers in this population; this is probably related to the low number of horses in this group that were selected by the referring veterinarians because of the suspicion of gastric lesions, due to either their clinical signs (recurrent colic, poor body condition score) or the exposure to other risk factors (new arrival on the premises, increased level of exercise in the weeks before the gastroscopy) [1,3,5,6,17].

Having a dental check less or more than 1 year before the examination was associated with an increased risk of both ESGD and EGGD. To the authors’ knowledge, this was never investigated in the literature, and its significance is not clear. This could be explained by the fact that most of the horses examined were included in these two groups compared to the others.

Results of the multiple logistic models show that signalment, management, and activity influence the development of gastric ulcers in the squamous mucosa. The pathophysiology of ESGD is associated with prolonged exposure of the mucosa to acid; its defense mechanisms are sparse, limited only to the keratinization of the epithelium, the tight junctions, high electrical resistance, and an osmophilic phospholipid surfactant-like layer. Acid injury, related to hydrochloric acid, short-chain fatty acids, lactic acid, and bile salts, all act synergistically to create a cascade of events that alters the surface of the mucosa, resulting in first hyperkeratosis, followed by sloughing with the formation of erosions and ulcers. Management and exercise have been demonstrated to result in prolonged exposure of the squamous mucosa to acid because of extended time spent without food, changes in abdominal pressure secondary to exercise, and isolation in the box without contact with other horses [5,33,38,41].

On the other hand, multiple logistic models for EGGD showed that only signalment was related to lesions of the glandular mucosa. Currently, the pathophysiology of the disease is not completely elucidated, but various studies have reported that management and dietary factors are not important for the development of EGGD [4,6,11,14]. The breakdown of normal protective factors is considered the primary mechanism behind alterations to the glandular mucosa. Stress and increased sensitivity to it have been associated with EGGD, and it has been demonstrated that horses positive for the disease have an increased response to ACTH stimulation compared to healthy ones [42,43]. This could explain why, in the population studied in our paper, signalment, and therefore, the subjective response of the horse to the environment and its stressors, is more important than management in the development of lesions in the glandular mucosa.

The presence of clinical signs, especially colic, and behaviors normally considered indicative of lesions (stable vices, yawning, teeth grinding, etc.) were not associated with ESGD or EGGD in this population. This is in line with current literature findings, where the presence of clinical signs is not indicative of gastric ulcers and the gold standard for diagnosis is gastroscopic examination [1,2,3,5,6,15].

This study has several limitations. Despite being asked, very few owners/caretakers were able to quantify the amount of supplementary feed or hay given to their horses. The volume of concentrate was generally quite low, even in horses exercising 5 to 6 times per week, not exceeding 0.5 kg/100 kg body weight. Hay was more variable, but at least 2 kg/100 kg body weight was given each day when the horses were not fed at libitum. Referring veterinarians, owners, and caretakers selected the animals for the endoscopies based on clinical signs. All subjects on each premises were supposed to be available for gastroscopy, but that was not always possible due to the demands of the shows in sport horses. This could have led to a selection bias of the animals based on their perceived importance or the presence of clinical signs. These, though, were not associated with ESGD or EGGD in this population.

## 5. Conclusions

In this cohort of 316 horses, the prevalence of ESGD was comparable to that reported in other studies [1,3,6], while EGGD was present in a lower percentage of horses compared to the literature [3,4,6]. The presence of ESGD was associated with the signalment of horses and their management, especially time and type of paddock, type of hay and supplementary feed administered, and time spent with the current owner/caretaker. Lesions of the squamous mucosa were also determined by the type of activity, with being used for breeding increasing the risk of ulcers in this part of the stomach compared to other types of exercise and time spent exercising during the week. EGGD, on the other hand, seems to be influenced by the signalment of the horses, with minimal effect on the management parameters evaluated with this questionnaire. These findings highlight the need for further studies about the presence of gastric ulcers in breeding animals and the importance of supporting gastric functions in horses not used for high levels of exercise, especially in mares and stallions, during the breeding season.

## Figures and Tables

**Table 1:** Responses to the questionnaire: for each answer, the number and percentage of the horses found positive and negative for ESGD and EGGD are reported.

Question		ESGD		EGGD	
	Total Number of Answers (Percentage)	Positive (Percentage)	Negative (Percentage)	Positive (Percentage)	Negative (Percentage)
**SECTION A: Signalment**					
**1. Age groups**					
Young	169/316 (53%)	72/169 (43%)	97/169 (57%)	25/169 (15%)	144/169 (85%)
Adult	124/316 (39%)	38/124 (31%)	86/124 (69%)	7/124 (6%)	117/124 (94%)
Old	23/316 (7%)	4/23 (17%)	19/23 (83%)	0/23 (0%)	23/23 (100%)
**2. Breed groups**					
Blood horses	149/316 (47%)	69/149 (46%)	80/149 (54%)	22/149 (15%)	127/149 (85%)
Saddlebred	144/316 (46%)	35/144 (24%)	109/144 (76%)	7/144 (5%)	137/144 (95%)
Baroque horses	14/316 (4%)	5/14 (36%)	9/14 (64%)	1/14 (7%)	13/14 (93%)
Coldblooded horses	2/316 (1%)	2/2 (100%)	0/2 (0%)	0/2 (0%)	2/2 (100%)
Ponies	7/316 (2%)	3/7 (43%)	4/7 (57%)	2/7 (29%)	5/7 (71%)
**3. Sex**					
Female	185/316 (58%)	69/185 (37%)	116/185 (63%)	14/185 (8%)	171/185 (92%)
Gelding	88/316 (28%)	27/88 (31%)	61/88 (69%)	10/88 (11%)	78/88 (89%)
Male	43/316 (14%)	18/43 (42%)	25/43 (58%)	8/43 (19%)	35/43 (81%)
**4. Temperament**					
Calm	238/316 (75%)	74/238 (31%)	164/238 (69%)	24/238 (10%)	214/238 (90%)
Nervous	78/316 (25%)	40/78 (51%)	38/78 (49%)	8/78 (10%)	70/78 (90%)
**5. How long have you been the owner/caretaker of your horse?**					
Since foal	51/316 (16%)	11/51 (22%)	40/51 (78%)	1/51 (2%)	50/51 (98%)
Less than 6 months	83/316 (26%)	36/83 (43%)	47/83 (57%)	8/83 (10%)	75/83 (90%)
6 months–1 year	31/316 (10%)	15/31 (48%)	16/31 (52%)	7/31 (23%)	24/31 (77%)
1–2 years	24/316 (8%)	11/24 (46%)	13/24 (54%)	3/24 (13%)	21/24 (87%)
3–5 years	73/316 (23%)	26/73 (36%)	47/73 (64%)	9/73 (12%)	64/73 (88%)
6–10 years	36/316 (11%)	12/36 (33%)	24/36 (67%)	4/36 (11%)	32/36 (89%)
More than 10 years	18/316 (6%)	3/18 (17%)	15/18 (83%)	0/18 (0%)	18/18 (100%)
**SECTION B: MANAGEMENT**					
**6. What kind of bedding do you use for your horse?**					
The horse does not live in a stable	91/316 (29%)	32/91 (35%)	59/91 (65%)	6/91 (7%)	85/91 (93%)
Shavings	66/316 (21%)	17/66 (26%)	49/66 (74%)	3/66 (5%)	63/91 (95%)
Straw	128/316 (41%)	58/128 (45%)	70/128 (55%)	19/128 (15%)	109/128 (85%)
Other (no bedding, rice hull, and paper)	31/316 (10%)	7/31 (23%)	24/31 (77%)	4/31 (13%)	27/31 (87%)
**7. What kind of paddock does your horse use?**					
No paddock available	124/316 (39%)	57/124 (46%)	67/124 (54%)	20/124 (16%)	104/124 (84%)
Sand	108/316 (34%)	39/108 (39%)	69/108 (64%)	7/108 (6%)	101/108 (94%)
Pasture	76/316 (24%)	15/76 (20%)	61/76 (80%)	5/76 (7%)	71/76 (93%)
Other (woodland and concrete)	8/316 (3%)	3/8 (38%)	5/8 (62%)	0/8 (0%)	8/8 (100%)
**8. How much time does your horse spend in the paddock?**					
No paddock available	123/316 (39%)	57/123 (46%)	66/123 (54%)	20/123 (16%)	103/123 (84%)
All day	120/316 (38%)	39/120 (32%)	81/120 (68%)	7/120 (6%)	113/120 (94%)
Stabled in the box during the night	73/316 (23%)	18/73 (25%)	55/73 (75%)	5/73 (7%)	68/73 (93%)
**9. How many times a day do you feed your horse (roughage and supplementary feed)?**					
Ad libitum	88/316 (28%)	26/88 (30%)	62/88 (70%)	4/88 (5%)	84/88 (95%)
2 times	94/316 (30%)	21/94 (22%)	73/94 (78%)	1/94 (1%)	93/94 (99%)
3 times	113/316 (36%)	52/113 (46%)	61/113 (54%)	19/113 (17%)	94/113 (83%)
More than 3 times	21/316 (7%)	15/21 (71%)	6/21 (29%)	8/21 (38%)	13/21 (62%)
**10. What kind of hay do you feed your horse?**					
Grass hay	269/316 (85%)	97/269 (36%)	172/269 (64%)	23/269 (9%)	246/269 (91%)
Alfalfa	25/316 (8%)	14/25 (56%)	11/25 (44%)	9/25 (36%)	16/25 (64%)
Grass hay supplemented only during winter	20/316 (6%)	2/20 (10%)	18/20 (90%)	0/20 (0%)	20/20 (100%)
Other (hay cobs)	2/316 (1%)	1/2 (50%)	1/2 (50%)	0/2 (0%)	2/2 (100%)
**11. What kind of supplementary feed do you give your horse?**					
Nothing	20/316 (6%)	3/20 (15%)	17/20 (85%)	2/20 (10%)	18/20 (90%)
Mixed	170/316 (54%)	50/170 (29%)	120/170 (71%)	7/170 (4%)	163/170 (96%)
Mixed + pelleted	83/316 (26%)	42/83 (51%)	41/83 (49%)	20/83 (24%)	63/83 (76%)
Oats only	1/316 (0%)	0/1 (0%)	1/1 (100%)	0/1 (0%)	1/1 (100%)
Pelleted	7/316 (2%)	3/7 (43%)	4/7 (57%)	0/7 (0%)	7/7 (100%)
Pressed	22/316 (7%)	14/22 (64%)	8/22 (36%)	1/22 (5%)	21/22 (95%)
Other (vegetables, bread, and bran)	13/316 (4%)	2/13 (15%)	11/13 (85%)	2/13 (15%)	11/13 (85%)
**SECTION C: ACTIVITY**					
**12. What kind of activity does your horse perform?**					
Nothing	64/316 (20%)	13/64 (20%)	51/64 (80%)	3/64 (5%)	61/64 (95%)
Breeding	99/316 (31%)	42/99 (42%)	57/99 (58%)	7/99 (7%)	92/99 (93%)
Trekking	30/316 (9%)	8/30 (27%)	22/30 (73%)	2/30 (7%)	28/30 (93%)
Jumping/Dressage/Eventing	64/316 (20%)	21/64 (33%)	43/64 (67%)	6/64 (9%)	58/64 (91%)
Endurance	14/316 (4%)	2/14 (14%)	12/14 (86%)	2/14 (14%)	12/14 (86%)
Riding lessons	15/316 (5%)	6/15 (40%)	9/15 (60%)	2/15 (13%)	13/15 (87%)
Other (driving, Western, and show)	30/316 (9%)	22/30 (73%)	8/30 (27%)	10/30 (33%)	20/30 (67%)
**13. How many days and for how long does your horse exercise each week?**					
No exercise	179/316 (57%)	63/179 (35%)	116/179 (65%)	10/179 (6%)	169/179 (94%)
1 time/week or less	30/316 (9%)	6/30 (20%)	24/30 (80%)	2/30 (7%)	28/30 (93%)
2–3 times/week or 2–3 h/week	35/316 (11%)	9/35 (26%)	26/35 (74%)	0/35 (0%)	35/35 (100%)
4–5 times/week or 4–6 h/week	19/316 (6%)	7/19 (37%)	12/19 (63%)	2/19 (11%)	17/19 (89%)
6–7 times/week or more than 6 h/week	53/316 (17%)	29/53 (55%)	24/53 (45%)	18/53 (34%)	35/53 (66%)
**14. How many shows/competitions does your horse attend in a month?**					
None	273/316 (86%)	100/273 (37%)	173/273 (63%)	25/273 (9%)	248/273 (91%)
1 or less	28/316 (9%)	10/28 (36%)	18/28 (64%)	5/28 (18%)	23/28 (82%)
2 or 3	15/316 (5%)	4/15 (27%)	11/15 (73%)	2/15 (13%)	13/15 (87%)
**15. How many times per month does your horse travel?**					
None	271/316 (86%)	97/271 (36%)	174/271 (64%)	24/271 (9%)	247/271 (91%)
1 or less	30/316 (9%)	11/30 (37%)	19/30 (63%)	5/30 (17%)	25/30 (83%)
2 or 3	15/316 (5%)	6/15 (40%)	9/15 (60%)	3/15 (20%)	12/15 (80%)
**16. Did your horse perform heavy exercise in the past (at least more than 2 years ago)?**					
None	226/316 (72%)	81/226 (36%)	145/226 (64%)	22/226 (10%)	204/226 (90%)
Endurance	11/316 (3%)	0/11 (0%)	11/11 (100%)	0/11 (0%)	11/11 (100%)
Show jumping	11/316 (3%)	2/11 (18%)	9/11 (82%)	0/11 (0%)	11/11 (100%)
Racing	62/316 (20%)	26/62 (42%)	36/62 (58%)	8/62 (13%)	54/62 (87%)
Historical horseracing	5/316 (2%)	4/5 (80%)	1/5 (20%)	2/5 (40%)	3/5 (60%)
Long distance trekking	1/316 (0%)	1/1 (100%)	0/1 (0%)	0/1 (0%)	1/1 (100%)
**SECTION D: HEALTH**					
**17. How long ago was your horse’s last dental checkup?**					
Never	189/316 (60%)	84/189 (44%)	105/189 (56%)	27/189 (14%)	162/189 (86%)
Less than 1 month	4/316 (1%)	2/4 (50%)	2/4 (50%)	0/4 (0%)	4/4 (100%)
Between 1 and 2 months	4/316 (1%)	0/4 (0%)	4/4 (100%)	1/4 (25%)	3/4 (75%)
Between 2 and 6 months	13/316 (4%)	6/13 (46%)	7/13 (54%)	3/13 (23%)	10/13 (77%)
Between 6 months and 1 year	42/316 (13%)	5/42 (12%)	37/42 (88%)	0/42 (0%)	42/42 (100%)
More than 1 year	64/316 (20%)	17/64 (27%)	47/64 (73%)	1/64 (2%)	63/64 (98%)
**18. Are you using any medications on your horse?**					
No	313/316 (99%)	113/313 (36%)	200/316 (64%)	32/313 (10%)	281/313 (90%)
Yes (inhaled corticosteroids, pergolide)	3/316 (1%)	1/3 (33%)	2/3 (67%)	0/3 (0%)	3/3 (100%)
**19. Did your horse show any colic signs while under your care?**					
No	289/316 (91%)	103/289 (36%)	186/289 (64%)	30/289 (10%)	259/289 (90%)
Yes	27/316 (9%)	11/27 (41%)	16/27 (59%)	2/27 (7%)	25/27 (93%)
**20. When was the last colic episode?**					
Never	289/316 (91%)	103/289 (36%)	186/289 (64%)	30/289 (10%)	259/289 (90%)
1–6 months ago	21/316 (7%)	9/21 (43%)	12/21 (57%)	2/21 (10%)	19/21 (90%)
7–12 months ago	1/316 (0%)	1/1 (100%)	0/1 (0%)	0/1 (0%)	1/1 (100%)
1–2 years ago	2/316 (1%)	0/2 (0%)	2/2 (100%)	0/2 (0%)	2/2 (100%)
More than 2 years ago	3/316 (1%)	0/3 (0%)	3/3 (100%)	0/3 (0%)	3/3 (100%)
**21. How severe were the colic signs, and what kind of treatment did the referring veterinarian administer?**					
No colic signs	289/316 (91%)	103/289 (36%)	186/289 (64%)	30/289 (10%)	259/289 (90%)
Low-intensity signs; no treatment required	8/316 (3%)	1/8 (13%)	7/8 (88%)	0/8 (0%)	8/8 (100%)
Low-intensity signs; medical treatment required (flunixin meglumine, N-butylscopolammonium bromide, fluid therapy, other)	11/316 (3%)	6/11 (55%)	5/11 (45%)	1/11 (9%)	10/11 (91%)
High-intensity signs; medical treatment required (flunixin meglumine, N-butylscopolammonium bromide, fluid therapy, other)	2/316 (1%)	0/2 (0%)	2/2 (100%)	0/2 (0%)	2/2 (100%)
High-intensity signs; surgical treatment required	0/316 (0%)	--	--	--	--
Recurrent colic signs	6/316 (2%)	3/6 (50%)	3/6 (50%)	1/6 (17%)	5/6 (83%)
**22. Does your horse have any stable vices (windsucking, weaving, etc.)**					
No	302/316 (96%)	106/302 (35%)	196/302 (65%)	31/302 (10%)	271/302 (90%)
Yes	14/316 (4%)	8/14 (57%)	6/14 (43%)	1/14 (7%)	13/14 (93%)
**23. Is your horse’s feeding behavior normal?**					
Yes	312/316 (99%)	112/312 (36%)	200/312 (64%)	31/312 (10%)	281/312 (90%)
No (dysorexia)	4/316 (1%)	2/4 (50%)	2/4 (50%)	1/4 (25%)	3/4 (75%)
**24. Is your horse showing changes in behavior (more agitated, quieter, etc.)?**					
No	312/316 (99%)	113/312 (36%)	199/312 (64%)	31/312 (10%)	281/312 (90%)
Yes	4/316 (1%)	1/4 (25%)	3/4 (75%)	1/4 (25%)	3/4 (75%)
**25. Is your horse showing “strange” behaviors (“playing with drinking water, yawning, etc.)?**					
No	311/316 (98%)	112/311 (36%)	199/311 (64%)	31/311 (10%)	280/311 (90%)
Yes	5/316 (2%)	2/5 (40%)	3/5 (60%)	1/5 (20%)	4/5 (80%)

**Table 2:** Odds ratios, confidence intervals, and p-values of the statistically significant simple logistic models for the presence of ESGD in this population of 316 horses enrolled.

Question	Odds Ratio	Confidence Interval	*p*-Value
**Age as number**	0.95	0.91–0.99	0.025
**Age groups**			
Young	reference		
Adult	0.60	0.36–0.97	0.037
Old	0.28	0.08–0.79	0.028
**Breed groups**			
Blood horses	reference		
Saddlebred	0.37	0.22–0.61	<0.001
Baroque horses	0.64	0.19–1.96	0.4
Coldblood horses	NA	NA	>0.9
Ponies	0.87	0.17–4.07	0.9
**Temperament**			
Calm	reference		
Nervous	2.33	1.39–3.94	0.001
**How long have you been the owner/caretaker of your horse?**			
Since foal	reference		
Less than 6 months	2.79	1.29–6.39	0.012
6 months–1 year	3.41	1.31–9.22	0.013
1–2 years	3.08	1.09–8.91	0.035
3–5 years	2.01	0.90–4.71	0.1
6–10 years	1.82	0.69–4.83	0.2
More than 10 years	0.73	0.15–2.73	0.7
**What kind of paddock does your horse use?**			
No paddock available	reference		
Grass	0.29	0.14–0.55	<0.001
Sand	0.66	0.39–1.12	0.13
Other	0.71	0.14–3.00	0.6
**How much time does your horse spend in the paddock?**			
No paddock available	reference		
All day	0.56	0.33–0.94	0.028
Only during daylight	0.38	0.20–0.71	0.003
**How many times per day do you feed your horse (roughage and supplementary feed)?**			
Ad libitum	reference		
2 times	0.69	0.35–1.33	0.3
3 times	2.03	1.14–3.70	0.018
More than 3 times	5.96	2.17–18.3	<0.001
**What kind of hay do you feed your horse?**			
Grass hay	reference		
Alfalfa	2.26	0.99–5.27	0.054
Grass hay supplemented only during winter	0.20	0.03–0.70	0.032
Other	1.77	0.07–45.2	0.7
**What kind of supplementary feed do you feed your horse?**			
No supplementary feed	reference		
Mixed	2.36	0.75–10.4	0.2
Mixed + pelleted	5.80	1.78–26.2	0.008
Oats only	0.00		>0.9
Pelleted	4.25	0.60–32.3	0.14
Pressed	9.92	2.44–52.8	0.003
Other	1.03	0.12–7.22	>0.9
**What kind of activity does your horse perform?**			
No exercise	reference		
Breeding	2.89	1.43–6.16	0.004
Endurance	0.65	0.09–2.81	0.6
Jumping/dressage/eventing	1.92	0.87–4.36	0.11
Riding lessons	2.62	0.76–8.67	0.12
Trekking	1.43	0.50–3.89	0.5
Other	10.8	4.07–31.3	<0.001
**How many days and for how long does your horse exercise each week?**			
No exercise	reference		
1 time/week or less	0.46	0.16–1.12	0.11
2–3 times/week or 2–3 h/week	0.64	0.27–1.40	0.3
4–5 times/week or 4–6 h/week	1.07	0.38–2.81	0.9
6–7 times/week or more than 6 h/week	2.22	1.20–4.17	0.012
**How long ago was your horse’s dental checkup?**			
Never	reference		
Less than 1 month	1.25	0.15–10.6	0.8
Between 1 and 2 months	0.00	--	>0.9
Between 2 and 6 months	1.07	0.33–3.34	>0.9
Between 6 months and 1 year	0.17	0.06–0.41	<0.001
More than 1 year	0.45	0.24–0.83	0.013

**Table 3:** Results of the multiple logistic models for the presence of ESGD.

Question	Odds Ratio	Confidence Interval	*p*-Value
**SECTION A: SIGNALMENT**			
**Age groups**			
Young	reference		
Adult	0.60	0.34–1.07	0.087
Old	0.45	0.10–1.65	0.3
**Breed**			
Blood horses	reference		
**Saddlebred**	**0.48**	**0.27–0.82**	**0.007**
Baroque horses	0.73	0.19–2.52	0.6
Coldblood horses	0.00	--	>0.9
Ponies	1.37	0.24–7.37	0.7
**Sex**			
Female	reference		
Gelding	1.06	0.57–1.97	0.9
Male	1.46	0.65–3.29	0.4
**Temperament**			
Calm	reference		
**Nervous**	**2.12**	**1.19–3.79**	**0.010**
How long have you been the owner/caretaker of your horse?			
Since foal	reference		
**Less than 6 months**	**2.53**	**1.07–6.29**	**0.038**
**6 months–1 year**	**3.16**	**1.13–9.11**	**0.029**
1–2 years	2.69	0.87–8.42	0.085
3–5 years	2.11	0.87–5.33	0.10
6–10 years	2.46	0.81–7.55	0.11
More than 10 years	1.47	0.24–7.45	0.7
**SECTION B: MANAGEMENT**			
**What kind of bedding do you use for your horse?**			
The horse does not live in a stable	reference		
**Shavings**	**0.26**	**0.07–0.89**	**0.035**
Straw	0.41	0.12–1.36	0.2
Others	0.32	0.08–1.20	0.1
**What kind of paddock does your horse use?**			
No paddock	Reference		
**Grass**	**0.26**	**0.10–0.63**	**0.004**
Sand	0.70	0.29–1.65	0.4
Other	0.40	0.07–1.96	0.3
**How many times a day do you feed your horse (roughage and supplementary feed?)**			
Ad libitum	reference		
2 times	0.94	0.29–3.08	>0.9
3 times	3.17	0.92–11.5	0.073
More than 3 times	4.30	0.78–24.7	0.10
**What kind of hay do you feed your horse?**			
Grass hay	reference		
Alfalfa	2.75	0.75–10.4	0.13
Grass hay supplemented only during winter	0.60	0.08–2.92	0.6
Other	1.42	0.04–50.6	0.8
**What kind of supplementary feed do you give your horse?**			
No supplementary feed	reference		
**Mixed**	**4.03**	**1.20–18.6**	**0.04**
Mixed + pelleted	3.52	0.94–17.2	0.08
Oats only	0.00	--	>0.9
**Pelleted**	**13.8**	**1.46–140**	**0.022**
**Pressed**	**15.6**	**3.01–104**	**0.002**
Other	1.19	0.11–10.4	0.9
**SECTION C: ACTIVITY**			
**What kind of activity does your horse perform?**			
Nothing	reference		
**Breeding**	**3.25**	1.51–7.42	**0.003**
Endurance	7.36	0.46–123	0.15
**Jumping/dressage/eventing**	**6.66**	1.37–38.6	**0.022**
**Riding lessons**	**8.13**	1.27–60.5	**0.030**
Trekking	9.86	1.55–73.6	0.019
**Other**	**22.2**	**4.69–142**	**<0.001**
**How many days and for how long does your horse exercise each week?**			
No exercise	reference		
**1 time/week or less**	**0.10**	**0.01–0.57**	**0.013**
2–3 times/week or 2–3 h/week	0.20	0.03–0.95	0.053
4–5 times/week or 4–6 h/week	0.43	0.07–2.39	0.3
6–7 times/week or more than 6 h/week	0.97	0.16–5.04	>0.9
**How many shows/competitions does your horse attend in a month?**			
None	reference		
1 or less	0.26	0.01–3.47	0.4
2–3 times per month	0.00	--	>0.9
**How many times per month does your horse travel?**			
None	reference		
1 time or less	2.27	0.20–54.1	0.5
2–3 times per month	0.00	--	>0.9
**Did your horse perform heavy exercise in the past (at least more than 2 years ago)?**			
None	reference		
Endurance	0.00		>0.9
Show jumping	0.41	0.06–1.73	0.3
Racing	0.90	0.46–1.75	0.8
Historical horseracing	5.32	0.44–129	0.2
Long distance trekking	0.00	--	>0.9
**SECTION D: HEALTH**			
**How long ago was your horse’s last dental check?**			
Never	reference		
Less than 1 month	1.18	0.12–12.3	0.9
Between 1 and 2 months	0.00	-	>0.9
Between 2 and 6 months	1.15	0.33–3.94	0.8
**Between 6 months and 1 year**	**0.12**	**0.04–0.33**	**<0.001**
**More than 1 year**	**0.39**	**0.19–0.76**	**0.007**
**Are you using any medications on your horse?**			
No	reference		
Yes	0.68	0.02–8.69	0.8
**Did your horse show any colic signs while under your care?**			
No	reference		
Yes	0.00	--	>0.9
**When was the last colic episode?**			
No	reference		
1–6 months ago	0.00	--	>0.9
7–12 months ago	0.00	--	>0.9
1–2 years ago	0.00	--	>0.9
More than 2 years ago	0.00	--	>0.9
**How severe were the colic signs, and what kind of treatment did the referring vet administer?**			
No	reference		
Low intensity; no treatment	0.00	--	>0.9
Low intensity; treatment	1.05	0.14–7.27	>0.9
High-intensity signs; medical treatment	0.00	--	>0.9
Recurrent colic signs	0.00	--	>0.9
**Does your horse have any stable vices (windsucking, weaving, etc.)?**			
No	reference		
Yes	2.09	0.56–8.80	0.3
**Is your horse’s feeding behavior normal?**			
Normal	reference		
Abnormal	1.45	0.14–16.4	0.7
**Is your horse showing changes in behavior (more agitated, quieter)?**			
No	reference		
Yes	0.72	0.06–9.63	0.8
**Is your horse showing “strange” behaviors (“playing” with drinking water, yawning, etc.)?**			
No	reference		
Yes	0.29	0.01–3.10	0.4
**SECTION D MODEL 1: COLIC SIGNS**			
**Did your horse show any colic signs while under your care?**			
No	reference		
Yes	0.00	--	>0.9
**When was the last colic episode?**			
Never	reference		
1–6 months ago	0.00	--	>0.9
7–12 months ago	0.00	--	>0.9
1–2 years ago	0.00	--	>0.9
More than 2 years ago	0.00	--	>0.9
**How severe were the colic signs, and what kind of treatment did the referring veterinarian administer?**			
No colic signs	reference		
Low intensity; no treatment	0.00	--	>0.9
Low intensity; treatment	1.50	0.19–12.4	0.7
High-intensity signs, medical treatment	0.00	--	>0.9
Recurrent colic signs	--	--	--
**SECTION D MODEL 2: CHANGES IN BEHAVIOR AND STABLE VICES**			
**Does your horse have any stable vices (windsucking, weaving, etc.)?**			
No	reference		
Yes	2.53	0.83–8.16	0.10
**Is your horse’s feeding behavior normal?**			
Normal	reference		
Abnormal	2.20	0.25–21.4	0.5
**Is your horse showing changes in behavior (more agitated, quieter, etc.)?**			
No	reference		
Yes	0.49	0.02–4.25	0.6
**Is your horse showing strange behaviors (“playing” with drinking water, yawning, etc.)?**			
No	reference		
Yes	0.84	0.10–5.49	0.9

Statistically significant parameters (*p* < 0.05) are in bold.

**Table 4:** Odds ratios, confidence intervals, and *p*-values of the statistically significant simple logistic models for the presence of EGGD in this population of 316 horses.

Risk Factor	Odds Ratio	Confidence Interval	*p*-Value
**Age as number**	0.93	0.86–0.99	0.04
**Age groups**			
Young	reference		
Adult	0.34	0.13–0.79	0.017
Old	0.00	--	>0.9
**Breed groups**			
Blood horses	reference		
Saddlebred	0.29	0.11–0.68	0.007
Baroque horses	0.44	0.02–2.41	0.4
Coldblood horses	0.00	--	>0.9
Ponies	2.31	0.32–11.5	0.3
**Sex**			
Female	reference		
Gelding	1.57	0.65–3.66	0.3
Male	2.79	1.05–7.04	0.033
**How long have you been the owner/caretaker of your horse?**			
Since foal	reference		
Less than 6 months	5.33	0.94–100	0.12
6 months–1 year	14.6	2.41–281	0.015
1–2 years	7.14	0.86–149	0.10
3–5 years	7.03	1.26–132	0.069
6–10 years	6.25	0.88–125	0.11
More than 10 years	0.00	--	>0.9
**What kind of paddock does your horse use?**			
No paddock available	reference		
Grass	0.37	0.12–0.95	0.055
Sand	0.36	0.14–0.85	0.027
Other	0.00	--	>0.9
**How much time does your horse spend in the paddock?**			
No paddock available	reference		
All day	0.32	0.12–0.75	0.013
Only during daylight	0.38	0.12–0.99	0.064
**How many times a day do you feed your horse (roughage and supplementary feed)?**			
Ad libitum	reference		
2 times	0.23	0.01–1.56	0.2
3 times	4.24	1.52–15.1	0.011
More than 3 times	12.9	3.56–54.5	<0.001
**What kind of hay do you feed your horse?**			
Grass hay	reference		
Alfalfa	6.02	2.32–15.0	<0.001
Grass hay supplemented only during winter	0.00	--	>0.9
Other	0.00	--	>0.9
**What kind of activity does your horse perform?**			
Nothing	reference		
Breeding	1.55	0.41–7.39	0.5
Endurance	3.39	0.41–22.7	0.2
Jumping/dressage/eventing	2.10	0.53–10.3	0.3
Riding lessons	3.13	0.38–20.8	0.2
Trekking	1.45	0.18–9.24	0.7
Other	10.2	2.80–48.8	0.001
**How many days and for how long does your horse exercise each week?**			
No exercise	reference		
1 time/week or less	1.21	0.18–4.89	0.8
2–3 times/week or 2–3 h/week	0.00	--	>0.9
4–5 times/week or 4–6 h/week	1.99	0.29–8.37	0.4
6–7 times/week or more than 6 h/week	8.69	3.77–21.1	<0.001
**How long ago was your horse’s last dental checkup?**			
Never	reference		
Less than 1 month	0.00	--	>0.9
Between 1 and 2 months	2.00	0.10–16.3	0.6
Between 2 and 6 months	1.80	0.39–6.34	0.4
Between 6 months and 1 year	0.00	--	>0.9
More than 1 year	0.10	0.01–0.46	0.022

**Table 5:** Results of the multiple logistic models for the presence of EGGD.

Risk Factor	Odds Ratio	Confidence Interval	*p*-Value
**PART A: SIGNALMENT**			
**Age groups**			
Young	reference		
**Adult**	**0.32**	**0.10–0.91**	**0.046**
Old	0.00	--	>0.9
**Breed groups**			
Blood horses	reference		
Saddlebred	0.38	0.14–0.97	0.054
Baroque horses	0.25	0.01–1.83	0.2
Coldblood horses	0.00	--	>0.9
Ponies	3.43	0.34–30.4	0.3
**Sex**			
Female	reference		
Gelding	2.38	0.89–6.25	0.077
**Male**	**5.08**	1.56–16.5	**0.006**
**Temperament**			
Calm	reference		
Nervous	0.69	0.23–1.82	0.5
**Time with the current caretaker**			
Since foal	reference		
Less than 6 months	7.63	1.21–150	0.069
**6 months–1 year**	**16.9**	**2.57–336**	**0.012**
1–2 years	9.57	1.05–210	0.065
3–5 years	8.45	1.35–166	0.055
**6–10 years**	**16.0**	**1.71–366**	**0.028**
More than 10 years	0.00	--	>0.9
**PART B: MANAGEMENT**			
**What kind of bedding do you use for your horse?**			
No stable	reference		
Shavings	0.48	0.05–4.03	0.5
Straw	0.50	0.08–3.47	0.5
Others	1.50	0.20–12.1	0.7
**What kind of paddock does your horse use?**			
No paddock available	reference		
Grass	0.52	0.11–2.04	0.4
Sand	0.52	0.11–1.99	0.4
Other	0.00	--	>0.9
**How many times a day do you feed your horse (roughage and supplementary feed)?**			
Ad libitum	reference		
2 times	0.16	0.01–1.83	0.2
3 times	2.35	0.31–17.5	0.4
More than 3 times	2.76	0.14–40.0	0.5
**What kind of hay do you feed your horse?**			
Grass hay	reference		
Alfalfa	4.63	0.70–45.1	0.13
Grass hay is added only during winter	0.00	--	>0.9
Other	0.00	--	>0.9
**What kind of supplementary feed do you give your horse?**			
No supplementary feed	reference		
Mixed	0.68	0.13–5.23	0.7
Mixed + pelleted	1.35	0.27–10.4	0.7
Oats only	0.00	--	>0.9
Pelleted	0.00	--	>0.9
Pressed	0.45	0.02–5.96	0.6
Other	0.75	0.03–12.6	0.8
**SECTION C: ACTIVITY**			
**What kind of activity does your horse perform?**			
Nothing	reference		
Breeding	1.37	0.33–6.83	0.7
Endurance	0.00	--	>0.9
Jumping/dressage/eventing	0.00	--	>0.9
Riding lessons	0.00	--	>0.9
Trekking	0.00	--	>0.9
Other	0.00	--	>0.9
**How many days and for how long does your horse exercise each week?**			
No exercise	reference		
1 time/week or less	--	--	>0.9
2–3 times/week or 2–3 h/week	--	--	>0.9
4–5 times/week or 4–6 h/week	--	--	>0.9
6–7 times/week or more than 6 h/week	--	--	>0.9
**How many shows/competitions does your horse attend in a month?**			
None	reference		
1 or less	0.00	--	>0.9
2–3 times per month	0.04	0.00–2.11	0.091
**How many times per month does your horse travel?**			
No	reference		
1 time or less	0.00	--	>0.9
2–3 times per month	13.6	0.49–383	0.081
**Did your horse perform heavy exercise in the past (at least more than 2 years ago)?**			
None	reference		
Endurance	0.00	--	>0.9
Show jumping	0.00	--	>0.9
Racing	0.92	0.25–2.96	0.9
Historical horseracing	1.07	0.08–13.2	>0.9
Long distance trekking	0.00	--	>0.9
**SECTION D: HEALTH**			
**How long ago was your horse’s last dental check?**			
Never	reference		
Less than 1 month	0.00	--	>0.9
Between 1 and 2 months	2.04	0.10–16.7	>0.9
Between 2 and 6 months	2.28	0.47–8.57	0.3
Between 6 months and 1 year	0.00	--	>0.9
**More than 1 year**	**0.05**	**0.00–0.34**	**0.02**
**Are you using any medications on your horse?**			
No	reference		
Yes	0.00	--	>0.9
**Did your horse show any colic signs while under your care?**			
No	reference		
Yes	0.00	--	>0.9
**When was the last colic episode?**			
No	reference		
1–6 months ago	0.00	--	>0.9
7–12 months ago	0.00	--	>0.9
1–2 years ago	0.00	--	>0.9
More than 2 years ago	0.00	--	>0.9
**How severe were the colic signs, and what kind of treatment did the referring vet administer?**			
No	reference		
Low intensity; no treatment	0.00	--	>0.9
Low intensity; treatment	0.16	0.00–7.20	0.3
High-intensity signs; medical treatment	0.00	--	>0.9
Recurrent colic signs	0.00	--	>0.9
**Does your horse have any stable vices (windsucking, weaving, etc.)?**			
No	reference		
Yes	0.61	0.03–3.96	0.7
**Is your horse’s feeding behavior normal?**			
Normal	reference		
Abnormal	1.70	0.04–24.4	0.7
**Is your horse showing changes in behavior (more agitated, quieter)?**			
No	reference		
Yes	4.69	0.13–168	0.3
Is your horse showing “strange” behaviors (“playing” with drinking water, yawning, etc.)?			
No	reference		
Yes	11.2	0.36–411	0.13
**SECTION D MODEL 1: COLIC SIGNS**			
**Did your horse show any colic signs while under your care?**			
No	reference		
Yes	0.00	--	>0.9
**When was the last colic episode?**			
No	reference		
1–6 months ago	0.00	--	>0.9
7–12 months ago	0.00	--	>0.9
1–2 years ago	1.16	--	>0.9
More than 2 years ago	4.51	--	>0.9
**How severe were the colic signs, and what kind of treatment did the referring vet administer?**			
No	reference		
Low intensity; no treatment	0.00	--	>0.9
Low intensity; treatment	0.56	0.02–16.1	0.7
High-intensity signs; medical treatment	0.00	--	>0.9
Recurrent colic signs	0.00	--	>0.9
**SECTION D MODEL 2: CHANGES IN BEHAVIOR AND STABLE VICES**			
**Does your horse have any stable vices (windsucking, weaving, etc.)?**			
No	reference		
Yes	0.57	0.03–3.31	0.6
**Is your horse’s feeding behavior normal?**			
Normal	reference		
Abnormal	2.38	0.10–21.8	0.5
**Is your horse showing changes in behavior (more agitated, quieter)?**			
No	reference		
Yes	2.38	0.10–21.8	0.5
**Is your horse showing “strange” behaviors (“playing” with drinking water, yawning, etc.)?**			
No	reference		
Yes	2.81	0.13–23.4	0.4

Statistically significant parameters (*p* < 0.05) are in bold.

## Data Availability

Data are available upon request from the authors.

## Supplementary Materials

The following supporting information can be downloaded at: https://www.mdpi.com/article/10.3390/ani14121806/s1, Table S1: Questionnaire administered to owners and caretakers of the horses included in this study.
