# Supplementary material for: Prevalence and Risk Factors for the Presence of Gastric Ulcers in Pleasure and Breeding Horses in Italy
Source: Animals (Basel). 2024 Jun 17;14(12):1806. doi: 10.3390/ani14121806 (PMC11201176; doi:10.3390/ani14121806)
Supplement: Supplementary file 1 [file animals-14-01806-s001.zip › animals-3039563-supplementary.pdf]

Date of the examination:
Name of the horse:
Name of the owner/caretaker:

SECTION A: Signalment

1. Age
2. Breed
3. Sex
    - Male
    - Female
    - Gelding
4. Temperament:
    - Calm
    - Nervous
5. How long have you been the owner/caretaker of your horse?
    - Since foal
    - Less than 6 months
    - 6 months-1 year
    - 1-2 years
    - 3-5 years
    - 6-10 years
    - More than 10 years

SECTION B: Management

6. What kind of bedding do you use for your horse?
    - The horse does not live in a stable
    - Shavings
    - Straw
    - Other (please explain)
7. What kind of paddock does your horse use?
    - The horse does not have a paddock available
    - Sand
    - Pasture
    - Other (please explain)
8. How much time does your horse spend in the paddock?
    - The horse does not have a paddock available
    - All day
    - It is stabled in box during the night
9. How many times a day do you feed your horse (roughage and supplementary feed)?
    - Ad libitum
    - 2 times
    - 3 times
    - More than 3 times
10. What kind of hay do you feed your horse?
    - Grass hay
    - Alfalfa
    - Grass hay supplemented only during winter
    - Other (please explain)
11. What kind of supplementary feed do you give your horse?
    - Nothing
    - Mixed

- Mixed+ pelleted
- Oats only
- Pelleted
- Pressed
- Other (please explain)

SECTION C: Activity

12. What kind of activity does your horse perform?
    - Nothing
    - Breeding
    - Trekking
    - Jumping/Dressage/
    - Eventing
    - Endurance
    - Riding lessons
    - Other (please explain)
13. How many days and for how long does your horse exercise each week?
    - No exercise
    - 1time/week or less
    - 2-3times/week or 2-3hrs/week
    - 4-5times/week or 4-6hrs/week
    - 6-7times/week or more than 6hrs/week
14. How many shows/competitions does your horse attend in a month?
    - None
    - 1 or less
    - 2 or 3
15. How many times per month does your horse travel?
    - None
    - 1 or less
    - 2 or 3
16. Did your horse perform heavy exercise in the past (at least more than 2 years ago)?
    - No
    - Yes (please explain)

SECTION D: Health

17. How long ago was your horse's last dental check up?
    - Never
    - Less than 1 month
    - Between 1 and 2 months
    - Between 2 and 6 months
    - Between 6 months and 1 year
    - More than 1 year
18. Are you using any medications on your horse?
    - No
    - Yes (please explain)
19. Did you horse show any colic signs while under your care?
    - No
    - Yes
20. When was the last colic episode?
    - Never
    - 1-6 months ago
    - 7-12 months ago

- 1-2 years ago
- More than 2 years ago

21. How severe were the colic signs and what kind of treatment did the referring veterinarian administer?
    - No colic signs
    - Low intensity signs, no treatment required
    - Low intensity signs, medical treatment required (flunixin meglumine, N-butylscopolammonium bromide, fluid therapy, other)
    - High intensity signs, medical treatment required (flunixin meglumine, N-butylscopolammonium bromide, fluid therapy, other)
    - High intensity signs, surgical treatment required
    - Recurrent colic signs
22. Does your horse have any stable vices (windsucking, weaving, etc)
    - No
    - Yes
23. Is your horse's feeding behaviour normal?
    - No
    - Yes
24. Is your horse showing changes in behaviour (more agitated, quieter, etc)?
    - No
    - Yes
25. Is your horse showing "strange" behaviours ("playing with drinking water, yawning,etc)?
    - No
    - Yes
